# The pharmacokinetics of levobupivacaine 0.5% after infraorbital or inferior alveolar block in anesthetized dogs

**DOI:** 10.3389/fvets.2022.1055231

**Published:** 2022-12-14

**Authors:** Matic Pavlica, Mojca Kržan, Ana Nemec, Tina Kosjek, Anže Baš, Alenka Seliškar

**Affiliations:** ^1^Small Animal Clinic, Veterinary Faculty, University of Ljubljana, Ljubljana, Slovenia; ^2^Department of Pharmacology and Experimental Toxicology, Faculty of Medicine, University of Ljubljana, Ljubljana, Slovenia; ^3^Department of Environmental Sciences, Jožef Stefan Institute, Ljubljana, Slovenia; ^4^Jožef Stefan International Postgraduate School, Ljubljana, Slovenia; ^5^Faculty of Education, University of Ljubljana, Ljubljana, Slovenia

**Keywords:** regional anesthesia, levobupivacaine, dexmedetomidine, pharmacology, animal experimentation, dogs

## Abstract

**Introduction:**

Data are lacking on the pharmacokinetic profile and safety of levobupivacaine (LB) used for regional anesthesia of the maxilla and mandibles in dogs.

**Methods:**

Infraorbital block (*n* = 10), inferior alveolar block (*n* = 10) or both infraorbital and inferior alveolar blocks (*n* = 10) were administered to dogs undergoing dental surgery under isoflurane anesthesia. The dose of LB was calculated as 0.11 ml/kg^2/3^ for the infraorbital block and 0.18 ml/kg^2/3^ for the inferior alveolar block. Blood samples were collected before and immediately after administration of the oral blocks, and 3, 4, 7, 12, 17, 32, 47, 62, 92, and 122 min thereafter. Quantification of LB in plasma was performed by LC-MS/MS.

**Results and discussion:**

The results are presented as median and interquartile range. In dogs in which all four quadrants of the oral cavity were desensitized with LB, the *C*_max_ was 1,335 (1,030–1,929) ng/ml, the *T*_max_ was 7 (4–9.5) min, and the AUC_(0 → 120)_ was 57,976 (44,954–96,224) ng min/ml. Plasma concentrations of LB were several times lower than the reported toxic concentrations, and no signs of cardiovascular depression or neurotoxicity were observed in any of the dogs, suggesting that the occurrence of severe adverse effects after administration of LB at the doses used in this study is unlikely.

## Introduction

Bupivacaine is a long-acting local anesthetic (LA); onset of action is 5–10 min after administration, and the duration of sensory blockade is 2–10 h (1). Levobupivacaine (LB) is the active l-isomer of bupivacaine and has similar effects to racemic bupivacaine but is less cardiotoxic (2). In humans, more than 97% of LB is bound to plasma proteins (albumin and α1-glycoprotein), and 95%−99% is metabolized by hepatic biotransformation. Most of the LB is excreted as inactive metabolites in the urine (71%) and feces (24%) (3).

In the only study describing the pharmacokinetics of LB for desensitization of the oral cavity in humans, 0.5% LB was administered near the maxillary and mandibular third molars in a total dose of 6.6 ml, equivalent to ~0.5 mg/kg. The maximum plasma concentration (*C*_max_) was 578 ± 101 ng/ml, the time to reach maximum plasma concentration (*T*_max_) was 21 ± 3 min, the clearance (Cl) was 465 ± 178 ml/min, and the area under the curve (AUC) was 1,371 ± 442 ng h/ml (4).

Systemic toxicity of LAs occurs with overdose or accidental intravenous (IV) administration. Signs of central nervous system (CNS) intoxication usually precede the onset of cardiovascular system (CVS) toxicity in awake animals and are difficult to detect during anesthesia (5). In rats anesthetized with isoflurane in oxygen, plasma LB concentrations at the onset of seizure activity, at first dysrhythmia, and at asystole were 10.0 ± 1.1, 21.2 ± 1.8, and 36.2 ± 6.5 μg/ml, respectively (6). In conscious sheep administered LB IV over 3 min, the arterial blood LB concentration at the onset of convulsive behavior was 16.3 ± 4.0 μg/ml (7). In anesthetized dogs, the cumulative dose which induced CVS collapse was 27.3 ± 2.0 mg/kg, and the plasma concentration was 22.7 μg/ml. The dosing regimen for LB was based on the manufacturer's assumed clearance rate (0.9 L/h/kg). It was assumed that after 12-min initial infusion 88% of the desired plasma concentration would be achieved, and that after the 12-min maintenance infusion 92% of the desired target concentration would be achieved (8).

Levobupivacaine is used in dogs off-label, and no data have yet been published on the pharmacokinetics of LB used to desensitize the oral cavity in dogs. Although no similar studies have been published in dogs, the pharmacokinetics of bupivacaine have been examined following other local blocks in dogs and cats (9–13). Because the reported toxic doses are all after IV administration of LB (6–8), plasma concentrations of LB would not be expected to be as high after perineural administration. This study aimed to gain detailed insight into the pharmacokinetics of LB used for regional anesthesia of the maxilla and mandibles in dogs. We investigated the pharmacokinetic profile and possible occurrence of adverse effects of LB, when administered to desensitize one or all four quadrants of the oral cavity at doses described by Pascoe (1), and whether the absorption rate of LB differs with respect to the site of application (infraorbital canal or submucosal deposition near the inferior alveolar nerve).

## Materials and methods

### Animals

The study protocol was reviewed and approved by the Institutional Ethics Committee for Animal Welfare of the Veterinary Faculty, University of Ljubljana (No. 8-10-2020/7, date of approval 7.1.2021), and formal written consent was obtained from dog owners before inclusion in the study. Thirty dogs scheduled for different dental procedures were included in the study. Only dogs without a history of endocrinologic, renal, or liver disease and without ongoing therapy with angiotensin-converting enzyme inhibitors, corticosteroids, or non-steroidal anti-inflammatory drugs were included in the study. Exclusion criteria also included brachycephalic dog breeds and dogs with a body weight of <10 kg. All dogs received a comprehensive oral exam with full-mouth charting and dental radiographs, followed by basic periodontal therapy performed with a sonic scaler and other dental procedure(s) as clinically indicated (single or multiple tooth extractions, endodontic procedure, gingivectomy, inclined plane placement, and partial coronal pulpectomy).

Only dogs which were in physical status I or II as assessed by the American Society of Anesthesiologists were considered eligible. Preanesthetic physical examination and hematologic and serum chemistry analyses were performed on all dogs and all blood parameters were within normal limits.

The dogs were assigned into groups according to whether they required a dental procedure in one quadrant (left or right infraorbital block, *n* = 10, or left or right inferior alveolar block, *n* = 10) or in all four quadrants (both infraorbital blocks and both inferior alveolar blocks, *n* = 10) of the oral cavity. Levobupivacaine 0.5% was administered as part of the standard anesthetic protocol into one or both infraorbital canals and/or into the submucosa near the mandibular foramen on one or both mandibles. The dose of LB was calculated as 0.11 ml/kg^2/3^ for the infraorbital block and 0.18 ml/kg^2/3^ for the inferior alveolar block (1).

### Study protocol

Food was withheld for no more than 12 h before anesthesia, and water was allowed *ad libitum* until the procedure. First, a 20-gauge catheter was aseptically placed in the cephalic vein to administer the drugs and fluids. The dogs were premedicated with methadone 0.2 mg/kg IV and 5 min later anesthesia was induced with propofol titrated until endotracheal intubation was achieved. Anesthesia was maintained with isoflurane in oxygen and the dogs were allowed to breathe spontaneously. They were placed in dorsal recumbency on a dental table covered with soft pads. If rectal temperature decreased below 37°C, forced-air warming (Bair Hugger, 3M, USA) was applied. Systolic arterial pressure was measured non-invasively with an ultrasonic Doppler flow monitor (Model 811, Parks Medical Electronics, USA). Heart rate, respiratory rate, end-tidal isoflurane concentration, end-tidal CO_2_ tension, and ECG (lead II) were monitored continuously with a multiparameter monitor (B105 Patient Monitor, GE Medical Systems Information Technologies Inc, USA). Body temperature was measured every 15 min with a rectal thermometer.

A 22-gauge catheter was aseptically placed in the dorsal pedal artery to collect blood samples for pharmacokinetic analyses of LB. Oral blocks were performed after the arterial catheter was placed. The infraorbital block was performed intraorally using a fine 42 mm 27-gauge needle (Sulzer Mixpac GmbH, Germany). The length of the infraorbital canal was estimated by measuring the distance from the distal root of the ipsilateral third premolar tooth, where infraorbital foramen was palpated to the junction of the zygomatic bone with the maxilla. The needle was inserted into the infraorbital canal to the length that corresponded to the estimated length of the infraorbital canal, as described previously (1), to avoid the risk of ocular injury (14). The inferior alveolar block was performed with extraoral technique, also with a fine 42 mm 27-gauge needle. First, the mandibular foramen was palpated with one hand inside the oral cavity on the medial aspect of the mandible, caudal and ventral to the ipsilateral last molar tooth. With the other hand, the needle was then advanced vertically on the most ventral surface of the mandible and guided medially as close to the bone as possible toward the foramen. With both techniques, the bevel of the needle was always oriented in the same direction as the nerve fibers, and aspiration was always applied before LB injection. All blocks were performed with an aseptic approach and by the same dentist (AN).

For rescue analgesia during anesthesia (if heart rate, respiratory rate, or systolic arterial pressure increased by more than 30% of basal values), fentanyl 2 μg/kg IV was used. Propofol 0.5–1 mg/kg IV was administered, and the inspired isoflurane concentration adjusted by 0.1% when a positive palpebral reflex was observed. Carprofen 4 mg/kg was administered IV when systolic arterial pressure was first measured above 80 mmHg. During the procedure, Ringer's lactate solution was infused with an infusion pump (Infusion Pump SK-600I Vet, Shenzhen Mindray Scientific Co. LTD, China) at a rate of 5 ml/kg/h. After the procedure, the fluids were administered at a rate of 2–6 ml/kg/h until discharge. Buprenorphine 0.02 mg/kg IV was administered as a single dose 4 h after methadone premedication or methadone 0.2 mg/kg IV was repeated every 4 h until discharge. The dogs were prescribed peroral carprofen 2 mg/kg/12 h for 2–7 days, and if more analgesia was required, a transdermal fentanyl patch 4 μg/kg/h was applied at the end of the procedure.

### Blood sampling

Blood (4 ml) was collected from the arterial catheter into tubes containing lithium heparinate prior to administration of oral blocks (basal values), immediately after administration of oral blocks, and 3, 4, 7, 12, 17, 32, 47, 62, 92, and 122 min thereafter. For each collection, 1 ml of blood was discarded from the catheter, the sample collected, and afterwards the catheter flushed with 4 ml of 0.9% NaCl. Blood samples were centrifuged immediately after collection at 1,500 *g* for 15 min at room temperature. Plasma samples were separated into aliquots and stored at −80°C until analysis.

### Laboratory analysis

Plasma samples were prepared for analysis by an Ostro 96-well plate (Waters Corp., Milford, MA, USA) for protein precipitation and phospholipid removal. Four microliter of the internal standard bupivacaine-d9 (Bupivacaine-d9 HCl, LGC/Toronto Research Chemicals, Canada) at a concentration of 2.5 μg/ml was added to the 50-μl plasma sample and was diluted with 0.1% formic acid to the volume of 150 μL. This mixture was transferred to a 2 ml well of the Ostro™ well plate before the addition of 450 μl 0.1% formic acid in acetonitrile. Samples were then aspirated three times with a multichannel pipette and placed onto a positive pressure processor (Waters Corp.) by setting the flow to 60 psi for 5 min. The eluate was dried under a gentle stream of nitrogen and then reconstituted in 1 ml of acetonitrile/0.1% formic acid mixture (3/7). Finally, the sample was filtered through a 0.2 μm syringe filter with a regenerated cellulose membrane (Phenomenex, Torrance, CA, USA) and analyzed by liquid chromatography coupled with tandem mass spectrometry (LC-MS /MS).

LC-MS/MS analysis was performed using a Nexera ultra-high-performance LC (Shimadzu Corp., Japan) coupled to a QTRAP 4500 MS/MS system (AB Sciex, Germany). Separation was achieved at room temperature using a 5 cm Ascentis Express C18 (Supelco, Bellefonte, PA, USA) column with 2 μm particle size and 2.1 mm internal diameter. The mobile phases were acetonitrile (A) and 0.1% formic acid (B). The flow rate was 0.3 ml/min and the injection volume was 1 μl. The gradient started at 70% B and was ramped down to 0% B within 3 min, and then maintained for the next 0.5 min. Finally, the gradient was increased back to 70% B and held for 1.5 min to allow the column to equilibrate. The mass spectrometer was operated under positive electrospray ionization at a source temperature of 600°C and in multiple reaction monitoring acquisition mode. The software for the data station was Analyst v1.6.3. For LB, transitions 289 > 140, 289 > 98, and 289 > 112 were monitored, with the first one used as the quantifying transition. For the internal standard LB-d9, transitions 298 > 149 and 198 > 101 were used to monitor retention time and process quality.

The limit of quantification (LOQ) of the method was 16 ng/ml and the limit of detection (LOD) was 6.0 ng/ml. The calibration range was from LOQ to 4.0 μg/ml, with a linear regression quotient >0.999. The quality of sample preparation and analysis were monitored through solvent blanks, process blanks, and quality control samples prepared by spiking the blank plasma with analyte and internal standard at the LOQ level. The accuracy determined for the QC samples was ±15% and precision was 7.3%. The method validation and all quality control procedures were in accordance with FDA guidelines for validation of bioanalytical methods (https://www.fda.gov/files/drugs/published/Bioanalytical-Method-Validation-Guidance-for-Industry.pdf).

### Pharmacokinetic analysis

The pharmacokinetic parameters were calculated using Graph Pad Prism software version 9.4.1. The *C*_max_ and *T*_max_ were determined directly from the raw data of individual measurements of LB concentrations from the blood samples of 30 animals. The area under the curve was calculated as AUC_(0 → 120)_ from the time 0 to the last quantifiable data point at 120 min using a linear trapezoid method. The data are presented as median and interquartile range.

### Statistical analysis

Normality of distribution of pharmacokinetic data was analyzed using the Shapiro-Wilk test. Given the mixture of normally and non-normally distributed data, values are expressed as median and interquartile range. To eliminate extraneous variables such as sex ratio, weight, and age as possible confounding variables, the Kruskall–Wallis test and Fisher's exact test were performed. Differences in pharmacokinetic parameters between groups were analyzed using the Kruskal–Wallis test with Dunn's *post-hoc* multiple comparison analysis. Statistical analysis was performed using Jamovi 2.0.0.0 (The Jamovi Project 2021, Australia) and Graph Pad Prism software version 9.4.1. Statistical significance was set at *p* < 0.05.

## Results

The average age of the dogs in this study was 72 (SD = 43) months and they weighed 25.2 (SD = 7.8) kg on average. There were no significant differences between groups in regard to weight *H*(2) = 0.194, *p* = 0.91 and age *H*(2) = 1.312, *p* = 0.52. Differences in sex ratio between groups were also not significant (*p* = 0.26, Fisher's exact test).

None of the dogs in this study required rescue analgesia with fentanyl during anesthesia. Six dogs in the LB IO group, seven dogs in the LB IA group, and six dogs in the LB ALL group received a propofol bolus and/or the isoflurane concentration was increased to deepen anesthesia in response to the return of a palpebral reflex that occurred when little or no stimulation was present. Propofol was also administered and isoflurane concentration increased when a positive palpebral reflex was observed due to a noxious stimulus that caused an increase in heart rate, respiratory rate, or systolic arterial pressure of <30% of basal values.

The volume of collected blood for pharmacokinetic analysis (4 ml × 12 = 48 ml) plus the volume of discarded blood (1 ml × 12 = 12 ml) totaled 60 ml. In the smallest dog, which weighed 11.2 kg, 60 ml of blood accounted for ~6.0% of the total blood volume. Blood loss due to the dental procedure was estimated by the number of blood-soaked swabs, where one fully soaked swab was equivalent to 10 ml of blood. Blood loss due to the dental procedure did not exceed 3% of total blood volume in any of the dogs. The packed cell volume was checked in all dogs before and after the procedure, and the values were always within the reference range.

No clinically evident adverse effects of the blocks were observed in any dog after the procedure.

### Pharmacokinetic analysis

**Figure 1:**
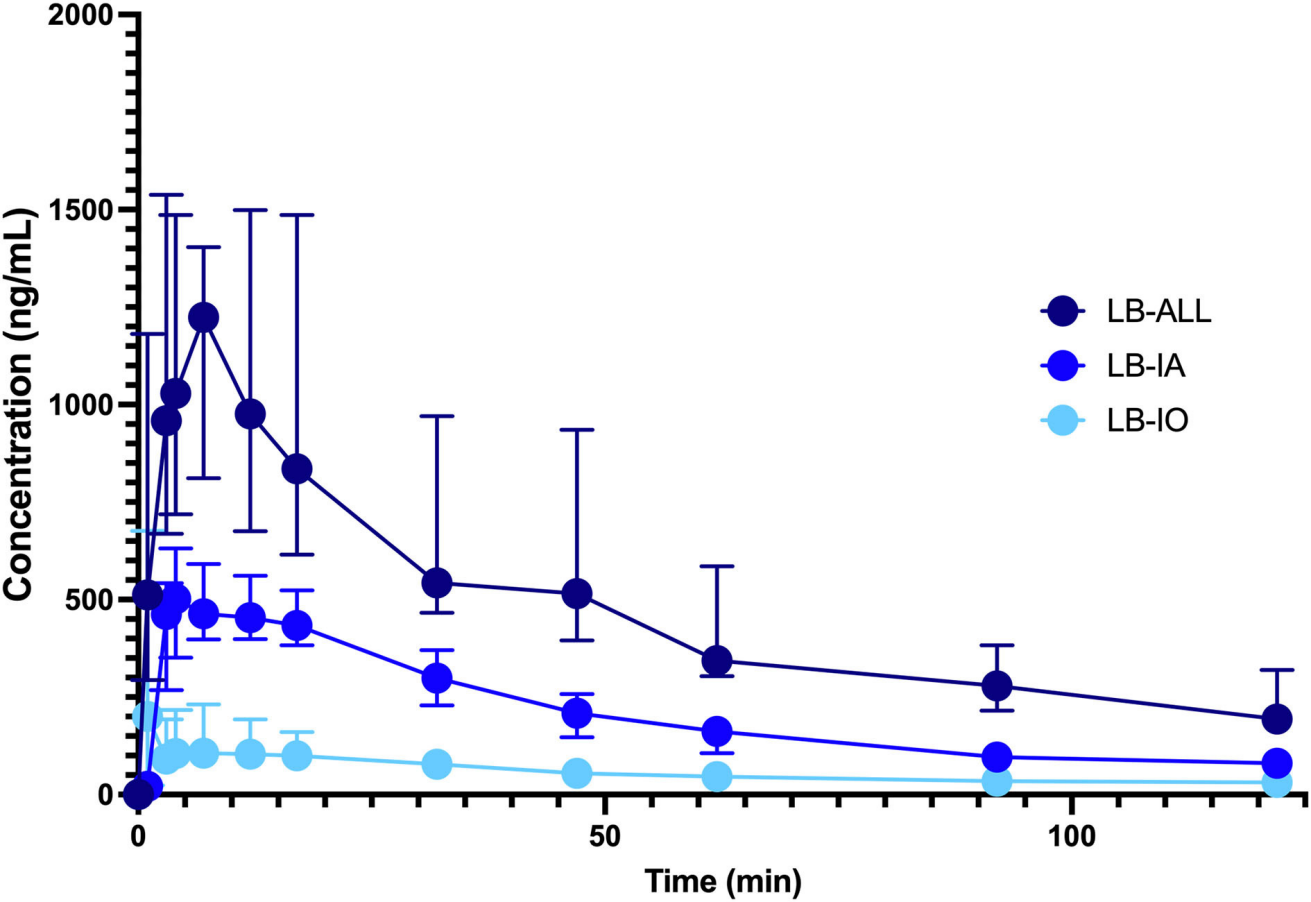
Concentration-vs.-time profile of LB 0.5% injected perineurally as a single infraorbital block (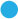, *n* = 10, LB IO) or single inferior alveolar block (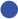, *n* = 10, LB IA group) or both infraorbital and inferior alveolar blocks (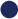, *n* = 10, LB ALL group). Dogs were premedicated with methadone intravenously, and anesthesia was induced with propofol and maintained with isoflurane in oxygen. The dose of LB was calculated as 0.11 ml/kg^2/3^ for the infraorbital block and 0.18 ml/kg^2/3^ for the IA block. The data are presented as median with interquartile range.

The calculated *C*_max_, *T*_max_ and AUC_(0 → 120)_ are presented in Table 1. Maximal plasma concentrations were determined in samples taken between 3 and 12 min (range 9 min LB IO) or 3 and 17 min (range 14 min LB IA and LB ALL). The *T*_max_ was the shortest in LB IA group, followed by LB IO and LB ALL groups. No significant difference in *T*_max_ was observed between groups. The *C*_max_ was the highest in LB ALL group, followed by LB IA and LB IO groups. The difference between LB ALL and the other two groups was significant. The same was observed with AUCs. The concentration-vs.-time profile of LB is presented in Figure 1.

**Table 1:** The calculated pharmacokinetic parameters of levobupivacaine.

	**LB-ALL**	**LB-IA**	**LB-IO**
Sample size, *n*	10	10	10
*C*_max_ (ng/ml)	1,335 (1,030–1,929)	601 (488–652)^*^	108 (94–242)^****^
*T*_max_ (min)	7 (4–9.5)	4 (3–9.5)	5.5 (3.75–8.25)
AUC_(0 → 120)_ (ng min/ml)	57,976 (44,954–96,224)	25,457 (19,200–28,872)^*^	6,595 (5,353–10,308)^****^

Values are presented as median with interquartile range,

* p < 0.05 and

**** p < 0.0001 significance Kruskal–Wallis test and Dunn's *post-hoc* test to LB-ALL.

AUC_(0 → 120)_, the area under the curve from time 0 to the last quantifiable data point at 120 min; C_max_, maximum plasma concentration; IA, inferior alveolar block; IO, infraorbital block; LB, levobupivacaine; T_max_, time to reach maximum plasma concentration.

## Discussion

This study confirmed that plasma concentrations of LB at the doses described by Pascoe (1) are several times lower than the reported toxic concentrations and that severe adverse effects of LB in terms of CNS and CVS toxicity are highly unlikely when used for regional anesthesia of the oral cavity in dogs anesthetized with isoflurane. The absorption rate of LB did not differ with respect to the site of application (infraorbital canal or submucosal deposition near the inferior alveolar nerve).

The cumulative dose and plasma concentrations of LB at the onset of CNS or CVS toxicity were studied in rats, sheep and dogs (6–8). The median total and free plasma concentrations required to produce cardiac collapse and median cumulative dose to collapse in anesthetized dogs administered LB IV were 22,700 ng/ml, 9,400 ng/ml, and 27.3 ± 2.0 mg/kg, respectively (8). The *C*_max_ in the LB ALL group in our study was 1,213 ± 69.8 ng/ml, which is approximately 19 times lower than the plasma concentration at the onset of CVS toxicity in anesthetized dogs administered LB intravenously (8), indicating that the occurrence of severe adverse effects is unlikely when it is used for regional anesthesia of the oral cavity in anesthetized dogs at a cumulative dose of up to 2 mg/kg.

In our study, the expected differences in *C*_max_ and AUC_(0 → 120)_ were observed between dogs given LB to desensitize all quadrants (LB ALL) and dogs given LB to desensitize only one quadrant (LB IO and LB IA). The *C*_max_ and AUC were consistent with the dose administered but not directly proportional to the dose. This observation may be due to the different route of administration of LB associated with possible differences in tissue vascularization of the infraorbital canal and submucosa near the inferior alveolar nerve. The different vasoactive properties of LB, leading to vasodilation at higher concentrations and vasoconstriction at lower concentrations, may also contribute to the observed differences (15).

Administration of LB into the infraorbital canal did not result in a shorter *T*_max_ compared with administration into the submucosa near the mandibular foramen. Data from the LB IO group were not normally distributed, whereas data from the LB IA group were normally distributed, making comparison between groups difficult. We suspect that interindividual differences in the width of the infraorbital canal and the pressure therein after administration of LB may have contributed to the non-normal distribution of the data in the LB IO group.

The dogs in our study were anesthetized with isoflurane; therefore, the pharmacokinetics of LB may differ from that in conscious animals. For example, sheep anesthetized with halothane were administered 125 mg LB IV over 3 min, and the blood concentration of LB was double that of the conscious sheep. The *T*_max_ was also longer in anesthetized sheep (16). Likewise, plasma concentrations of lidocaine administered 2 mg/kg IV differed significantly between conscious and isoflurane-anesthetized cats, as evidenced by more than two-fold higher values of extrapolated plasma drug concentration at time 0 (C_p0_) in anesthetized cats (17).

Although the clinical signs of CNS toxicity could be masked by anesthesia, the cardiovascular depression caused by inhalational anesthetics could be exacerbated due to the high plasma concentration of LAs. On the other hand, anesthesia could protect against the toxic effects of LAs and possibly death, because in the LB toxicity study all sheep anesthetized with halothane survived compared with conscious sheep (18). In our study, only non-invasive CVS monitoring was performed, and no signs of clinically significant cardiovascular depression or neurotoxicity were observed in any group of dogs.

The major limitation of this clinical study is the non-standardized dental procedure. In some dogs, the procedure was completed before the last blood sample was taken, and it would be ethically unacceptable to prolong anesthesia solely because of the requirements of this study. In this case the remaining blood samples were collected from non-anesthetized dogs, which might have affected the pharmacokinetics of LB (19). Because the total volume of blood drawn from each dog was 60 ml, no dogs weighing <10 kg were included in this study.

In conclusion, our study confirmed that (1) plasma concentrations of LB are several times lower than reported toxic concentrations when LB is used for regional anesthesia of the oral cavity in isoflurane-anesthetized dogs at doses described by Pascoe (1), and (2) the absorption rate of LB did not differ with respect to the site of application.

## Data availability statement

The raw data supporting the conclusions of this article will be made available by the authors, without undue reservation.

## Ethics statement

This study was approved by the Animal Welfare Committee at Veterinary Faculty University of Ljubljana, Slovenia (approval number 8-10-2020/7, approval date 7 January 2021). All procedures complied with the relevant Slovenian (Animal Protection Act UL RS, 43/2007) and European regulations. Formal written informed consent was obtained from dog owners before participation in the study. Their participation was voluntary, and they could withdraw from the study at any time. They were informed that all data and photographic material would be published without disclosure of the identity of the dog or its owner.

## Author contributions

This study was conceived and designed by MP, MK, AN, and AS. Data were analyzed by MK and AB. All authors contributed to study conduct, manuscript preparation, and approved the submitted version.

## References

[1] Pascoe PJ. Anesthesia and pain management. In:Verstraete FJM Lommer MJ Arzi B, editors. Oral and Maxillofacial Surgery in Dogs and Cats, 2nd ed New York, NY: Elsevier (2020), p. 22–43. ISBN: 9780702076756. 10.1016/B978-0-7020-7675-6.00013-9

[2] McLeod GA Burke D. Levobupivacaine. Anaesthesia. (2001) 56:331–41. 10.1046/j.1365-2044.2001.01964.x 11284819

[3] Foster RH Markham A. Levobupivacaine: a review of its pharmacology and use as a local anesthetic. Drugs. (2000) 59:551–79. 10.2165/00003495-200059030-00013 10776835

[4] Fawcett JP Kennedy JM Kumar A Ledger R Kumara GM Patel MJ. Comparative efficacy and pharmacokinetics of racemic bupivacaine and S-bupivacaine in third molar surgery. J Pharm Pharm Sci. (2002) 5:199–204. 12207874

[5] Leone S Di Cianni S Casati A Fanelli F. Pharmacology, toxicology, and clinical use of new long acting anesthetics, ropivacaine and levobupivacaine. Acta Biomed. (2008) 79:92–105. 18788503

[6] Ohmura S Kawada M Ohta T Yamamoto K Kobayashi T. Systemic toxicity and resuscitation in bupivacaine, levobupivacaine, or ropivacaine-infused rats. Anesth Analg. (2001) 93:743–8. 10.1097/00000539-200109000-00039 11524350

[7] Chang DH Ladd LA Wilson KA Gelgor L Mather LE. Tolerability of large-dose intravenous levobupivacaine in sheep. Anesth Analg. (2000) 91:671–9. 10.1213/00000539-200009000-00033 10960398

[8] Groban L Deal DD Vernon JC L James RL Butterworth J. Cardiac resuscitation after incremental overdosage with lidocaine, bupivacaine, levobupivacaine, and ropivacaine in anesthetized dogs. Anesth Analg. (2001) 92:37–43. 10.1097/00000539-200101000-00008 11133597

[9] Franquelo C Toledo A Manubens J Valladares JE Cristòfol C Arboix M. Pharmacokinetics and pharmacologic effects of the S(-) isomer of bupivacaine after intravenous and epidural administration in dogs. Am J Vet Res. (1999) 60:832–5. 10407475

[10] Bubenik L Hosgood G Barker BM Serra V Stout R. Estimated plasma bupivacaine concentration after single dose and eight-hour continuous intra-articular infusion of bupivacaine in normal dogs. Vet Surg. (2007) 36:783–91. 10.1111/j.1532-950X.2007.00337.x 18067620

[11] Benito J Monteiro B Beaudry F Steagall P. Efficacy and pharmacokinetics of bupivacaine with epinephrine or dexmedetomidine after intraperitoneal administration in cats undergoing ovariohysterectomy. Can J Vet Res. (2018) 82:124–30. 29755192 PMC5914079

[12] Shilo-Benjamini Y Pypendop BH Newbold G Pascoe PJ. Plasma bupivacaine concentrations following orbital injections in cats. Vet Anaesth Analg. (2017) 44:178–82. 10.1111/vaa.12388 27216369

[13] Garbin M Benito J Ruel HLM Watanabe R Monteiro BP Cagnardi P. Pharmacokinetics of bupivacaine following administration by an ultrasound-guided transversus abdominis plane block in cats undergoing ovariohysterectomy. Pharmaceutics. (2022) 14:1548. 10.3390/pharmaceutics14081548 35893804 PMC9331386

[14] Pumphrey SA Reader RC Rosenstein DS Mouser PJ Wetmore LA. Iatrogenic ocular trauma associated with infraorbital block performed for rhinoscopy in a cat: case report and preliminary imaging findings. JFMS Open Rep. (2021) 7:20551169211011456. 10.1177/20551169211011456 33996139 PMC8108087

[15] Newton DJ McLeod GA Khan F Belch JJ. Vasoactive characteristics of bupivacaine and levobupivacaine with and without adjuvant epinephrine in peripheral human skin. Br J Anaesth. (2005) 94:662–7. 10.1093/bja/aei095 15722384

[16] Copeland SE Ladd LA Gu XQ Mather LE. The effects of general anesthesia on whole body and regional pharmacokinetics of local anesthetics at toxic doses. Anesth Analg. (2008) 106:1440–9. 10.1213/ane.0b013e31816ba541 18420858

[17] Thomasy SM Pypendop BH Ilkiw JE Stanley SD. Pharmacokinetics of lidocaine and its active metabolite, monoethylglycinexylidide, after intravenous administration of lidocaine to awake and isoflurane-anesthetized cats. Am J Vet Res. (2005) 66:1162–6. 10.2460/ajvr.2005.66.1162 16111153

[18] Copeland SE Ladd LA Gu XQ Mather LE. The effects of general anesthesia on the central nervous and cardiovascular system toxicity of local anesthetics. Anesth Analg. (2008) 106:1429–39. 10.1213/ane.0b013e31816d12af 18420857

[19] Groban L Deal DD Vernon JC James RL Butterworth J. Ventricular arrhythmias with or without programmed electrical stimulation after incremental overdosage with lidocaine, bupivacaine, levobupivacaine, and ropivacaine. Anesth Analg. (2000) 91:1103–11. 10.1213/00000539-200011000-00011 11049891

